# Case Report: TRNT1 related autoinflammatory syndrome in a patient with primary ciliary dyskinesia

**DOI:** 10.3389/fimmu.2026.1778403

**Published:** 2026-07-01

**Authors:** Simona Di Gennaro, Francesca Della Casa, Angelica Petraroli, Melissa Borrelli, Maria Alessio, Francesca Orlando, Roberta Naddei

**Affiliations:** 1 Department of Translational Medical Science, Pediatric Rheumatology, University of Naples Federico II, Naples, Italy; 2 Department of Translational Medical Science, Division of Clinical Immunology, University of Naples Federico II, Naples, Italy; 3 Department of Translational Medical Sciences, Pediatric Pulmonology, University of Naples Federico II, Naples, Italy; 4 Department of Pediatrics, Azienda Ospedaliera di Rilievo Nazionale (AORN) Santobono Pausilipon, Naples, Italy

**Keywords:** autoinflammatory disease, case report, etanercept, genetic disorders, primary ciliary dyskinesia, TRNT1 syndrome

## Abstract

We report the case of a young woman with primary ciliary dyskinesia (PCD) who was also diagnosed with tRNA nucleotidyl transferase 1 (TRNT1)-related autoinflammatory syndrome, characterized by recurrent episodes of fever and arthralgia beginning at age 16. To our knowledge, this is the first documented case of homozygosity for the c.1246A>G variant in the TRNT1 gene. The patient had a milder clinical phenotype than previously reported cases with the same variant in a compound heterozygous state. Etanercept administration effectively controlled autoinflammatory manifestations, consistent with prior literature, and no adverse safety events were observed, despite the elevated risk of infectious pulmonary complications due to concurrent PCD. This case underscores the importance of considering autoinflammatory disease in patients presenting with relevant clinical features, even when manifestations are mild or have a delayed onset.

## Introduction

Systemic autoinflammatory diseases (SAIDs) comprise an expanding group of disorders. They are driven by dysregulation of the innate immune system and result in recurrent episodes of systemic inflammation. In many cases, SAIDs have a strong genetic basis, caused by pathogenic variants in a single gene. However, they may also arise from polygenic or multifactorial mechanisms. Environmental factors contribute to phenotypic variability. This complexity underscores the need for novel approaches to better define the relationship between molecular alterations and phenotypic manifestations (1, 2).

Mutations in the gene encoding tRNA nucleotidyl transferase 1 (TRNT1) have been associated with clinical phenotypes of varying severity. This wide spectrum includes a severe multiorgan syndrome named “Congenital sideroblastic anaemia with immunodeficiency, fever, and developmental delay (SIFD)”, or milder phenotypes characterized by autoinflammatory manifestations, moderate hematological and immunological abnormalities, or retinitis pigmentosa (3).

Our 19-year-old patient, affected by primary ciliary dyskinesia (PCD), presented a homozygous mutation in the *TRNT1* gene, resulting in a mild phenotype characterized by recurrent episodes of fever and arthralgias, associated with a significant increase in inflammatory markers.

## Case description

Herein, we report the case of a 19-year-old girl with PCD who was referred to our Centre with a history of recurrent episodes of fever and arthralgias. Her medical history began at 2 days of age, when she underwent surgical repair for intestinal malrotation and jejunal atresia. At age 10, due to chronic sinusitis, recurrent pneumonia, and bronchiectasis, she underwent an extensive respiratory assessment that showed a pathological nasal nitric oxide level (51 nl/minute) and reduced ciliary beat motility on high-speed digital video microscopy, with normal ultrastructural axoneme on transmission electron microscopy. Genetic testing revealed pathogenic biallelic mutations in the Dynein Axonemal Heavy Chain 5 (DNAH5) gene: a maternally inherited mutation (c.8314C>T) and a paternally inherited mutation (c.7914insA). Therefore, PCD was diagnosed, and respiratory follow-up and treatment were initiated.

At age 16, the patient developed recurrent episodes of fever, associated with arthralgias and, less frequently, joint swelling (**Table 1**). Whole-body magnetic resonance imaging showed mild joint effusions in the hips, left knee, ankles, and subtalar joints. The febrile episodes responded well to short courses of corticosteroids. At the age of 19, she was referred to our Centre. Clinical examination was unremarkable, except for wheezing, mild facial dysmorphisms, including brittle and sparse hair, and slender limbs. No signs of arthritis were detected. The lung disease was well controlled with inhaled therapy and respiratory physiotherapy, with normal respiratory function.

**Table 1:** Comparison between the clinical features of the present patient and the main manifestations of PCD and TRNT1 deficiency.

Patient main clinical features	TRNT1 deficiency	PCD syndrome	Laboratory and/or imaging findings
Constitutional symptoms: recurrent fever and reticuloendothelial activation	Typical	Absent	Increase of inflammatory markers (SAA, CRP)/mild splenomegaly
Facial dysmorphisms	Common	Absent	
Musculoskeletal symptoms	Possible	Absent	Increase of inflammatory markers (SAA, CRP); Whole-Body MRI: slight joints effusion
Immunohematological abnormalities	Typical	Absent	Normocytic normochromic anaemia; IgA 37 mg/dl; Lymphocyte CD19+ IgD+CD27+: 6%
Recurrent respiratory infections	Possible due to immunodeficiency	Typical	Episodes of sinusitis and pneumonia confirmed radiologically

SAA, serum amyloid A; CRP, C-reactive protein; MRI, magnetic resonance imaging; Ig, immunoglobulin.

Laboratory investigations showed normocytic anaemia, with normal iron indices, and a marked increase in acute-phase reactants, particularly serum amyloid A (SAA, 587 mg/l), a few days after a febrile episode. An isolated splenomegaly was detected on abdominal ultrasound.

A next-generation sequencing panel for SAIDs, comprising analysis of 138 genes, was performed, revealing the homozygous pathogenic mutation c.1246A>G (p.Lys416Glu) in the TRNT1 gene. No abnormalities were observed on the peripheral blood smear. Ophthalmological examination and echocardiogram were normal, as was the audiometric test. At immunological evaluation, the patient showed a normal response to vaccines and normal levels of immunoglobulin M (IgM) and IgG, with a slight decrease in IgA. The study of lymphocyte subsets showed a normal CD4/CD8 ratio (2.5) and a mild reduction in IgM memory B lymphocytes, with normal counts of naïve B lymphocytes and class-switched memory B lymphocytes (**Table 1**). Despite a mild immunohematological phenotype, characterized by slight B-cell impairment without sideroblastic anaemia, the recurrent febrile episodes associated with joint involvement and elevated inflammatory markers were consistent with TRNT1-related SAID, together with the subtle dysmorphic features.

**Figure 1:**
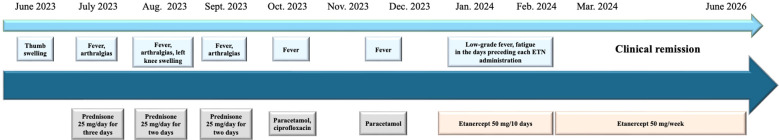
Timeline of the patient’s clinical course and treatment management.

Treatment with Etanercept (ETN) was initiated to achieve remission of the autoinflammatory manifestations, based on previous evidence supporting its efficacy in TRNT1-related SAID (4–8). Given the potential risk of respiratory exacerbations related to PCD, ETN was started at a reduced dosing schedule of 50 mg every 10 days. Fever and arthralgia episodes improved, and SAA decreased (**Figure 1**). However, in the days preceding each ETN administration, the patient frequently developed low-grade fever and fatigue. Therefore, the ETN regimen was intensified to a weekly dose of 50 mg, which resulted in complete clinical and laboratory remission. The patient also reported improvement in respiratory symptoms, including reduced sputum production and the absence of respiratory exacerbations. To date, she has been treated with ETN for 2 years, during which no further autoinflammatory episodes or safety events, including significant pulmonary exacerbations, have been observed.

## Discussion

Currently, only about sixty cases of *TRNT1*-related SAID have been reported. This disorder was first associated with SIFD (9), an autosomal recessive disease with severe multi-organ involvement that often results in death within the first decade of life, but over the years a wide phenotypic heterogeneity has been described (3). Patients at the severe end of the disease spectrum present with severe anaemia, immunodeficiency, and developmental delay, with a significant mortality rate (4). By contrast, a sizeable proportion of patients with TRNT1 deficiency exhibit a milder phenotype, including non-syndromic retinitis pigmentosa, mild hematologic abnormalities, or autoinflammatory manifestations with elevated inflammatory markers, the latter referred to as TRNT1-related SAID (4–7, 10–12). In line with this phenotypic spectrum, our patient presented with a mild phenotype, characterized by recurrent fever, subtle joint inflammatory symptoms, and increased acute-phase reactants, all of which responded well to corticosteroids. The recurrent nature of the clinical episodes, together with elevated inflammatory markers and the effectiveness of corticosteroids, prompted consideration of a diagnosis of SAID in this young woman, despite the concomitant condition of PCD and the late onset of symptoms. Consistent with TRNT1 deficiency, our patient also exhibited subtle dysmorphic features and a slight B-cell impairment. To our knowledge, this is the first reported case of TRNT1-related SAID with such a mild phenotype and adolescent-onset disease.

Notably, our patient carried the homozygous pathogenic missense mutation c.1246A>G (p.Lys416Glu) in the TRNT1 gene, which has previously been reported in six other patients (4, 7, 8, 13). In all these cases, however, the variant occurred in a compound heterozygous state, as summarized in **Table 2**. Compared with our patient, those patients exhibited a more severe phenotype, characterized by very early disease onset and generally associated with severe developmental delay, severe anaemia, hearing loss and cataracts, and immunodeficiency in addition to autoinflammatory manifestations (4, 7, 8, 13). It has been shown that TRNT1 enzyme activity, depending on genotype, correlates with disease severity. Complete TRNT1 enzyme deficiency is lethal early in development, whereas severe missense variants that retain partial residual enzymatic activity are associated with early-onset SIFD. In contrast, milder missense variants are generally associated with less sever phenotypes (14) It could be hypothesized that, in our case, homozygosity for the c.1246A>G (p.Lys416Glu) variant may result in lower protein instability compared with the compound heterozygous state, in which two different mutant protein structures coexist, thereby leading to a milder clinical phenotype. However, this hypothesis remains speculative and requires confirmation through further studies.

**Table 2:** Main clinical manifestations of patients carrying the c.1246A>G (p.Lys416Glu) variant in the TRNT1 gene, including the present patient.

Patient	Allele 1	Allele 2	Age at onset (months)	Clinical predominant features						
				Fever	Developmental delay	Immunodeficiency	Sideroblastic anemia	Ocular disease/hearing loss	Musculoskeletal symptoms	Other
1	c.1246A>G	c.1245_1246insA	2	P	P	P	P	P	P	A
2	c.938delT	c.1246A>G	1	P	P	P	P	P	P	GHD
3	c.608G>A	c.1246A>G	4	P	P	P	P	P	P	GHD
4	c.1056 + 1G > A	c.1246A > G	8	P	P	P	P	P	P	A
5	c.1056 + 1G >A	c.1246A > G	4	P	P	P	A	P	P	A
6	c.1246A> G	c.495_498del	6	A	A	A	P	P	A	Panniculitis
7 Present patient	c.1246A> G	c.1246A> G	16 years	P	A	A	A	A	P	A

GHD, Growth hormone deficiency; P, Present; A, Absent.

Our patient achieved excellent control of inflammatory signs and symptoms after starting ETN. The decision to initiate ETN was based on previously reported cases of TRNT1-related SAID in the literature. Among approximately 60 reported patients with TRNT1 deficiency, eleven have been treated with ETN, showing good control of musculoskeletal symptoms and systemic inflammation (4–8). ETN exerts its therapeutic effect by inhibiting Tumor Necrosis Factor alpha (TNF-α). It has been hypothesized that, in TRNT1 deficiency, TNF-α may be overproduced as a consequence of misfolded protein accumulation driven by tRNA dysregulation associated with TRNT1 mutations (4, 15). This mechanism could explain the effectiveness of ETN in controlling autoinflammatory manifestations in TRNT1-related SAID.

Patients receiving biologic agents are considered at increased risk of infection. Notably, after ETN initiation, the patient did not experience an increased frequency of respiratory exacerbations, despite the concomitant diagnosis of PCD. To date, few case reports have described the use of TNF-α inhibitors in patients with both rheumatologic and lung disease, and no significant safety concerns have been reported (16–19). Moreover, a meta-analysis of 1,434 children with juvenile idiopathic arthritis found that anti-TNF therapy was associated with only a slight, non-significant increase in the incidence of infections (20). Interestingly, the patient also reported clinical improvement in respiratory symptoms after ETN initiation. However, whether this finding was directly related to TNF-α inhibition remains uncertain.

In conclusion, our case highlights the importance of considering an autoinflammatory disease in adult patients presenting with suggestive clinical manifestations, even in the presence of concomitant diseases. To our knowledge, this is the first reported case of homozygosity for the c.1246A>G variant. Our patient exhibited a milder phenotype than previously described cases carrying the same variant in a compound heterozygous state, suggesting a possible downstream cascade effect, whereby protein instability may be greater when two different mutant protein structures coexist. However, further studies and functional assays are required to clarify the genotype–phenotype correlation. In our patient, ETN proved effective in controlling the autoinflammatory manifestations, consistent with previous reports, and was not associated with safety events, despite the higher risk of infectious pulmonary complications related to the concomitant diagnosis of PCD. Notably, the patient also reported improvement in respiratory symptoms since initiation of anti-TNF-α treatment.

## Data Availability

The raw data supporting the conclusions of this article will be made available by the authors, without undue reservation.
